# Seed sourcing for climate‐resilient grasslands: The role of seed source diversity during early restoration establishment

**DOI:** 10.1002/ece3.10756

**Published:** 2023-11-21

**Authors:** Jessica Lindstrom, Marissa Ahlering, Jill Hamilton

**Affiliations:** ^1^ Department of Biological Sciences North Dakota State University Fargo North Dakota USA; ^2^ The Nature Conservancy Minneapolis Minnesota USA; ^3^ Department of Ecosystem Science and Management Pennsylvania State University University Park Pennsylvania USA

**Keywords:** grassland restoration, plant community establishment, seed mix, seed source, species richness

## Abstract

Restoration advocates for the use of local seed in restoration, but theory suggests that diverse seed sources may enhance genetic diversity and longer term evolutionary potential within restored communities. However, few empirical studies have evaluated whether species and genetic diversity within species impacts plant community composition following restoration. The goal of this research is to compare the effects of single and multi‐sourced seed mix treatments on plant community diversity following restoration. Species establishment, abundance, and diversity were compared following two restoration seed mix treatments created to include 14 species commonly used in grassland restoration. We compared the application of seed mixes designed using a single population per species with those containing five populations per species across sites in Minnesota and South Dakota, United States. Early plant establishment and richness mostly reflected non‐seeded species across both sites, although seeded species established at a slightly higher rate in year two following restoration. At the South Dakota site, community composition largely reflected changes associated with establishment across the growing season as opposed to seed mix treatment. This contrasted with the Minnesota site, where community composition appeared to be strongly influenced by seed mix treatment. While there is some evidence seed mix treatment may be influencing the emergent community across sites, spatial heterogeneity across the Minnesota restoration site likely influenced diversity in early emergence over that of seed mix treatment. Indeed, varying land‐use history across both sites likely contributed to differences in species composition observed at this early stage of the restoration. This suggests that seed mix treatment may have limited impact on early post‐restoration emergence diversity relative to the importance of land‐use history. However, future monitoring will be needed to evaluate whether the impact of seed mix treatment on community composition changes over time.

## Graphical abstract

Seed source diversity within restoration seed mixes may be used as a proxy to assess the role of population genetic variation within species to restoration. Increasing seed source diversity within seed mixes may be necessary to ensure community resilience to changing conditions, but long‐term monitoring of restorations following application of seed mixes is needed. No matter the seed mix, land‐use history will influence what species readily establish following restoration and should be considered when planning a restoration.

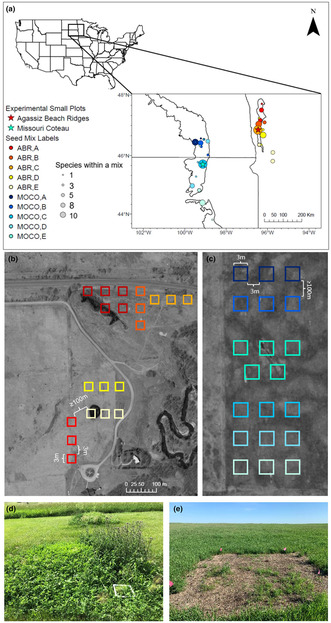

## 1 INTRODUCTION

Globally, native grasslands remain one of the most critically imperiled ecosystems requiring active restoration (Hamilton et al., 2020; Hoekstra et al., 2005). Throughout the North American Great Plains, up to 87% of historical grassland habitat has been lost primarily to agricultural conversion (Comer et al., 2018; Hoekstra et al., 2005; Samson et al., 2010). This has led to substantial fragmentation and isolation across remnant prairie habitats. Where grasslands remain, they are prone to invasion by non‐native species and the evolutionary consequences of isolation, which may reduce diversity and species richness (DiAllesandro et al., 2013; Haddad et al., 2015). To increase grassland hectares, land managers are attempting to reseed sites with seed mixes that will establish, adapt to change, resist invasion, and persist over time. However, the role of regional genetic diversity within species seed mixes to community composition following restoration has received limited attention.

A major aim of grassland restoration is to re‐establish functional plant species diversity to ensure key ecosystem services are maintained following restoration (Barr et al., 2017; Montoya et al., 2012). To ensure ecosystem health and the maintenance of productive plant communities, this includes creating seed mixes that reflect not only species diversity but also genetic variation within species (Brudvig, 2011; Tilman et al., 1996, 1997). Evolutionary theory emphasizes the important role that genetic diversity within seed mixes may have to restoration success over time (McKay et al., 2005). Species‐rich seed mixes create communities that may be resilient to (i) changes in nutrient availability (Craven et al., 2016), (ii) competition from non‐natives (Funk et al., 2008; Norland et al., 2013; Oakley & Knox, 2013; Yurkonis et al., 2012), and (iii) climate change (Isbell et al., 2015). In addition, genetic variation provides the raw material that natural selection may act upon and is needed for species to respond to environmental change (McKay et al., 2005; Zeldin et al., 2020). However, despite the importance of genetic diversity within species to restoration success, few studies have quantified its role to restoration outcomes (Hamilton et al., 2020). To ensure plant communities persist over time and respond to environmental change, there is a need to consider both the role of species and genetic diversity within species to restoration outcomes.

Genetic diversity within species is required alongside species rich seed mixes to ensure preservation of evolutionary potential. The combination of species diversity and genetic diversity within species can influence community composition during establishment and longer term (Larson et al., 2013). Diversity at these two scales can impact short‐term response to the environment and competition with local seed banks (Grman et al., 2013). During the first few years following restoration it is expected that communities will be largely dominated by non‐seeded weedy species typically found within the soil seed bank (Bakker et al., 2003). For example, when comparing an ongoing prairie restoration to multiple remnant prairies, Martin et al. (2005) observed more non‐native species present within the restoration, with the overall proportion of non‐natives ranging from 236% to 413% higher in the restoration relative to remnant sites. Thus, monitoring early establishment of seeded relative to non‐seeded species may be important to predicting how long‐term plant community composition may change. Particularly as early benchmarks of seeded species' emergence may provide an indication of overall restoration success (Brudvig & Catano, 2021; Gonzalez et al., 2013; Piotrowski et al., 2023). Despite the potential importance of early establishment to long‐term restoration success, this phase is often overlooked in favor of evaluating restorations after they have been established for several years.

Current strategies to design seed mixes often advocate a “local is best” approach (Broadhurst et al., 2008; McKay et al., 2005). This approach assumes that local seed sources will have the greatest fitness in the restored environment relative to non‐local sources (Kawecki & Ebert, 2004). While there is evidence of local adaptation for some plant species (Hereford, 2009; Leimu et al., 2010), the degree or scale of “local” adaptation when observed is often unknown (McKay et al., 2005). If seeds are sourced locally for restoration from small, isolated populations, an individual seed source may not have the requisite genetic variation needed to adapt to environmental change (Davis et al., 2005; Etterson & Shaw, 2001). Genetic variation may be reduced through random fluctuations in small, isolated populations via genetic drift (Reed & Frankham, 2003), reduced connectivity, or gene flow (Durka et al., 2017; Hamilton & Eckert, 2007). Furthermore, to conserve species' evolutionary potential requires genetic variation, suggesting that the addition of non‐local sources that may include regional genetic variation is important (Di Santo et al., 2022; Kawecki & Ebert, 2004; Volk et al., 2022). Regional provenancing of seeds from multiple sources may provide the genetic variation needed to ensure adaptability is maintained over time within a seed mix (Hofner et al., 2022). To ensure seed mixes used in restoration maintain optimal evolutionary potential, seed sourcing strategies that maintain genetic diversity will be needed (Volk et al., 2022). Ultimately, accounting for the role evolutionary forces play in the maintenance of diversity will aid in designing seed mixes that will increase restoration success (Bucharova et al., 2016; Hamilton et al., 2020).

We compared plant community composition following restoration using single‐ and multi‐source seed mixes to evaluate the role intraspecific diversity played in community establishment over time. We used seed collected from five unique populations for each of 14 different species as a proxy for creating regional genetic diversity within a seed mix. We expected that increasing the number of unique regional seed sources per species within a seed mix would lead to increased species richness and emergence diversity following restoration relative to the use of a single seed source per species (Bucharova et al., 2018). Overall, we predicted greater intraspecific diversity within seed mixes would lead to greater species diversity in restored plant communities. This research empirically evaluates the role of intraspecific diversity to the early emergent plant community following restoration, providing a baseline understanding of the role of diversity across scales to restoration establishment.

## 2 METHODS

### 2.1 Seed collection

**FIGURE 1:**
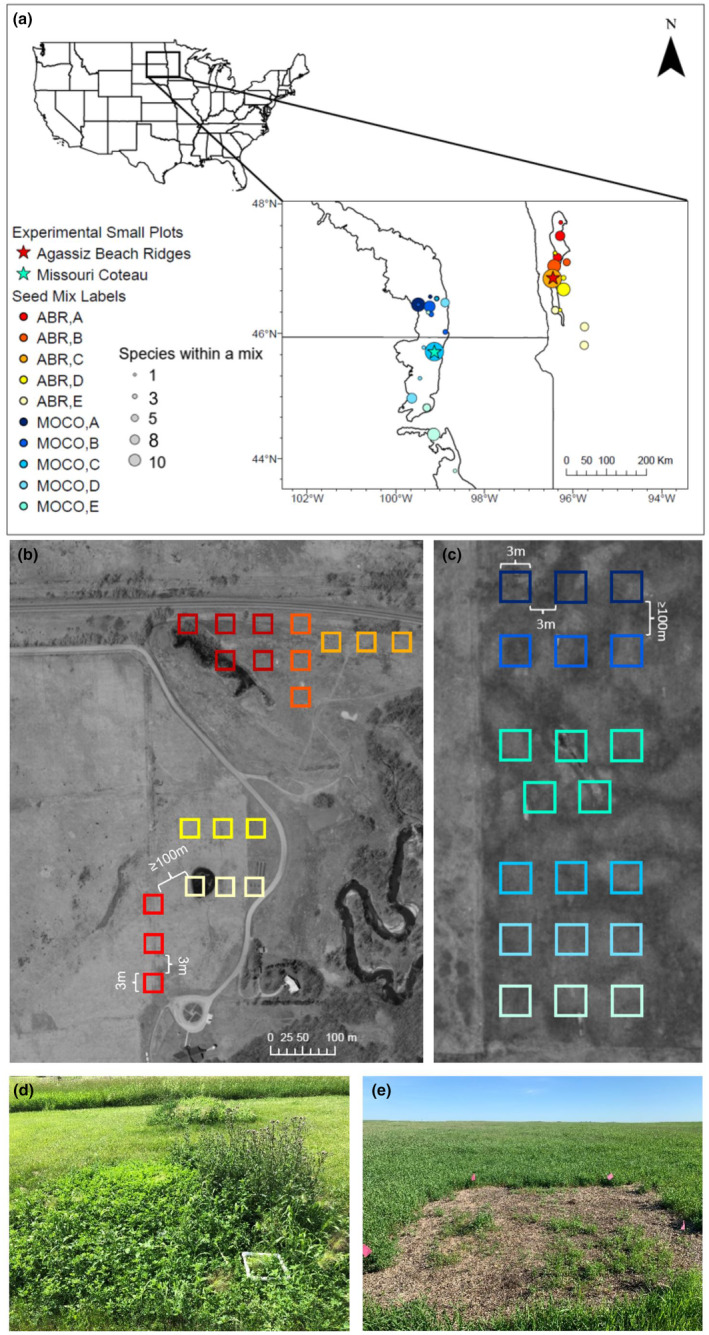
(a) Seed collection sites for seed mix treatments for the Missouri Coteau in North Dakota and South Dakota (blues) and northwestern MN (reds) regions, respectively. Colors represent individual seed mixes, and proportional symbols indicate the number of species sourced from a single site that were used within a seed mix. Stars indicate experimental site locations. (b) MN experimental plots layout at the Regional Science Center in Glyndon, MN. (c) SD experimental plots layout at the Samuel H. Ordway Memorial Preserve in Leola, SD. Colors correspond to seed treatment, single source treatments include three replicate plots and the five‐source treatment includes five replicate plots. (d) MN single source plot layout during July first year data collection. (e) SD single source plot layout during June first year data collection.

Between June and October of 2019, seeds from 12 forb and two grass species were collected from remnant native prairies throughout much of the Northern Great Plains of the United States (Table S1). These 14 species were chosen because they are widely distributed throughout the Northern Great Plains and are commonly used in regional restoration seed mixes (e.g., Kurtz, 2013; Smith, 2010). To control for potential dominance of warm‐season grasses within seed mixes and to increase establishment of sown forbs, species included were weighted more heavily toward forb species (Dickson & Busby, 2009; McCain et al., 2010; Norland et al., 2013). A minimum of five populations per species were collected from each of the Missouri Coteau region of North and South Dakota and from the northwestern prairie region of Minnesota in the United States (Table S1, Figure 1). Populations were classified as distinct if they were geographically separated by at least one mile. In northwestern Minnesota, distance between seed source populations ranged from 3 to 215 km and pairwise distances between the restoration site and seed source ranged from 2 to 129 km. Within the Missouri Coteau region, distance between seed source locations ranged from 2 to 312 km, and pairwise distance between the restoration site and seed sources ranged from 3.5 to 214 km.

Seeds were hand‐harvested as they ripened, with seed harvested on a site multiple times throughout the growing season following Bureau of Land Management seed harvesting guidelines. Individual maternal seed heads were sampled at least 3 feet apart within populations to reduce potential relatedness. For species with multiple seed heads, no more than 30% of available seed per seed head were collected.

### 2.2 Seed mix preparation

Following harvest, seeds were dried at room temperature for a minimum of 2 weeks and then transferred to 4°C dry storage for 7 months to maintain viability. Seeds were cleaned using several species‐specific approaches. Large seeds were stripped by hand, smaller seeds separated using sieves, *Hesperostipa comata* seed awns were trimmed during the drying process to limit tangling, and seeds of *Solidago rigida*, *Helianthus maximiliani*, and *H. pauciflorus* were mechanically cleaned and separated using a fractionating aspirator test model at the USDA Agricultural Research Center in Fargo, ND.

Seed weight was calculated for each population of each species (Mettler Toledo, ML503T/00) to estimate population‐specific number of seeds to include in restoration seed mixes using a seeds per gram conversion (Table S2). To ensure consistency across seed mix treatments and replicates, the number of seeds per species included in the restoration mix was calculated based on the population with the lowest seed weight (g). In addition, for *Artemisia frigida*, *H. pauciflorus*, and *S. rigida*, the number of seeds used in the seed mixes was reduced by 0.9%, 3.5%–7.0%, and 4.4%–6.0% of the lowest seed weight respectively. This was done to ensure these species were not overrepresented in seed mixes, as they exhibit competitive characteristics and can dominate their respective communities (Table S2).

Restoration seed mixes were designed using the same species across the two sites with the exceptions of *A. frigida*, *Anemone cylindrica*, and *Schizachyrium scoparium*, which were collected and planted exclusively in the northwestern Minnesota Agassiz Beach Ridges region (Minnesota site, MN), and *Ratibida columnifera*, *H. comata*, *Bouteloua gracilis*, which were collected and planted exclusively in the Missouri Coteau region (South Dakota site, SD). Five seed mixes were designed using a single seed source population for each species within each of the two restoration regions (MN and SD). For the single seed source mixes, species were sourced from populations spanning different geographic locations as all species did not co‐occur at a single location (Table S1). Given the different source locations for the different seed mixes (seed mix: A, B, C, D, and E), populations from different species were largely combined based on latitude. This was done to minimize potential impacts associated with latitudinal variation in phenology. Thus, seed mix “A” identifies populations largely sourced from northern latitudes and “E” identifies population sources from more southern latitudes within each region of assessment (Dunnell & Travers, 2011; Olsson & Agren, 2002) (Table S1, Figure 1). In addition to five single seed source mixes, one multiple‐source seed mix was designed for each region (seed mix: ABCDE). The multi‐source seed mix used the same number of seeds per species as the single‐source mix; however, each individual species' contribution to the mix was divided evenly across five different population sources. Thus, for both single and multi‐source seed mixes the proportion of seed used per species was the same. In this way, the ratio of species present within the single source and multi‐source was maintained across seed mixes for direct comparison. Vermiculite (Vigoro) was added to all final seed mixes in a 1:1 ratio (Shaw et al., 2020), a common method to increase seed to soil contact during planting and thus increase probability of emergence.

### 2.3 Seed viability

Seeds that remained unused in designing restoration seed mixes from the Minnesota site were sent to the South Dakota State University Seed Testing Laboratory to assess seed viability. Unused seed samples from the South Dakota site were not available for seed viability testing. Seed viability tests evaluated the total viability of individual species when grown under ideal laboratory growth conditions to induce germination. Viability tests reported the percentage of seed that germinated, defined as the total number of individuals emerged per seeds planted, percentage of hard seed, defined as dormant due to a water impervious seedcoat, and dormant seed, which is defined as viable but not germinable due to a physical or physiological condition (SDSU Seed Testing Laboratory; https://www.sdstate.edu/sites/default/files/file‐archive/2021‐07/Seed‐Testing‐Lab.pdf).

### 2.4 Experimental restoration site preparation

During May and June of 2019, experimental restoration sites were identified and prepared in the Agassiz Beach Ridges region of northwestern Minnesota (MN) and the Missouri Coteau region of South Dakota (SD). The northwestern Minnesota restoration site within the Agassiz Beach Ridges region was established at the Minnesota State University Moorhead Regional Science Center (MN, 46.872, −96.452) in Glyndon, MN. Portions of this site were once abandoned agricultural fields dominated by smooth brome (*Bromus inermis*) adjacent to remnant mesic prairie, owned and maintained by Buffalo River State Park. Between 1962 and 2015, another portion of this site was maintained as the Ponderosa Golf Course. However, following transfer of ownership in 2015 it has had limited management and only limited mowing for maintenance. Due to site and space limitations, both areas of this site were used to establish the experimental restoration plots. The Missouri Coteau region restoration site was established on the Samuel H. Ordway Prairie Preserve (SD, 45.704, −99.086) in South Dakota, which has been owned and managed by The Nature Conservancy (TNC) since 1978. Prior to TNC ownership, this site was used as a smooth brome grass/alfalfa production pasture for cattle. Since TNC's ownership, this site has been maintained for hay production every other year.

In 2019, the MN site was prepared by placing landscape cloth over experimental restoration plots to remove existing vegetation and limit potential establishment and competition with the existing seedbed prior to seeding. In fall 2019, prior to seeding, the SD site was treated with glyphosate herbicide (Roundup®, 3%–4% concentration) within each plot to reduce competition with existing weedy vegetation during establishment the following spring. Additionally, all plots had a second glyphosate treatment in early May 2020 to further reduce encroachment from smooth brome.

At each site, 20 3 × 3 m experimental restoration plots were established. This included five different single source seed treatment plots each replicated three times (*n* = 15), and one multi‐seed source treatment replicated five times (*n* = 5). To limit the potential for dispersal and gene flow between plots, a 3 m barrier was maintained between replicated plots within each seed treatment, and a minimum 100 m buffer was maintained between each single‐ and multi‐source seed treatment plot.

### 2.5 Planting experimental restoration treatments

To establish restoration treatments, tarps were removed from the plots at the MN site, and litter was raked and hand‐weeded in April 2020 at both sites to expose the seed bed. Following this, each plot was broadcast seeded and then raked again to increase seed–soil contact. For both sites, five times the total commonly recommended seeding rate of ~5 kg (11 pounds) of seeds per acre were applied to increase the probability of emergence success (Rowe, 2010). Higher seeding rates were applied, as higher rates have previously been associated with increased establishment and diversity (Barr et al., 2017; Sheley & Half, 2006). An agri‐fab push lawn roller was used to increase seed to soil contact and enhance the probability of germination. To limit potential carryover of seeds between treatments, the roller was rinsed and dried between each application. Finally, each plot received a one‐time watering treatment. Throughout the growing season, plot maintenance included weekly barrier mowing around each plot. In July, mid‐season mowing was performed at both sites to increase light availability and reduce competition with non‐seeded species (Kaul & Wilsey, 2021; Maron & Jefferies, 2001). Plots were mowed at the maximum adjustable height setting (12.7 cm), and all trimmings were removed.

### 2.6 Data collection

Each plot was visited once per month at both sites between June and September of 2020 and 2021 to assess plant community composition. A 0.2 m^2^ quadrat was placed at each of the four cardinal corners and center of each plot at least 2.5 cm away from the plot edge to estimate community composition. To minimize the impact of edge effects, quadrats were not placed directly at the edges of each plot. For all species present in the quadrat, we counted the number of individuals present. Unidentifiable individuals were marked with unique toothpicks and photographed for later identification. There were two unknown species at the MN site and three at the SD site that did not match seeded species and could not be identified. These species were uniquely labeled as unknowns and included in diversity calculations as unique non‐seeded species. Total percentage cover of dead vegetation and percentage bare soil cover were also assessed visually within the quadrat. All data collected in non‐summary form is made available on Github (https://github.com/lindstroje/Lindstrom_EE/tree/main/Data).

### 2.7 Statistical analysis

To infer plant productivity and assess plant community composition following restoration, species diversity indices, including richness, evenness, and abundance, were monitored across sites over time (Table S3). We evaluated differences in community composition across the first (2020) and second (2021) year following restoration based on seed mix treatment at each of our restoration sites. Species richness was defined as the total number of species present across all five quadrats sampled per replicate, and abundance was calculated as the total number of individuals present per species across quadrats. We also analyzed the total number of unique species and the number of seeded species that established within seed treatments for replicated plots. To evaluate our communities regardless of planted or non‐seeded species status, we calculated Shannon's Diversity Index (H′) for each seed treatment and each replicate plot from June to September across years (Magurran, 2004). All statistical analyses were performed in R (Version 4.0.2).

To evaluate differences between community compositions for different seed mix treatments across the two separate years of data collection, we performed permutational ANOVAs (PERMANOVAS). PERMANOVA uses species distance matrices based on community composition for seed treatments using the *adonis* function in the package “vegan” to quantify differences between individual and multiple‐source seed treatments over time (Oksanen et al., 2022). This approach is particularly appropriate where species presence data are zero skewed and do not conform to normality (Anderson, 2006). However, PERMANOVA tests may be sensitive to data dispersion associated with unequal sample size and site heterogeneity. Data dispersion between seed treatments, reflected as beta diversity, may indicate that changes to community composition reflect seed treatments or sampling effects associated with within treatment replication heterogeneity or additional dispersion facts, such as planting site location (Anderson, 2006; Anderson & Walsh, 2013). Accounting for data dispersion was particularly important for this experiment, as it provided a means to account for unbalanced design between seed mix treatments, where single seed source treatments were replicated three times and multi‐source treatments were replicated five times. This would potentially influence beta‐diversity estimates per seed mix treatment. We evaluated within treatment homogeneity using a PERMADISP analysis, leveraging the *betadispr* function in the package “vegan” to test if replicates differed in their dispersion (Anderson, 2006; Anderson & Walsh, 2013). The number of permutations for each test was set to 999. To quantify the relationship between seed mix treatment and spatial distances associated with planting location and community composition differences over time, Mantel tests were performed in the R package “MDMR” (McArtor, 2022). Comparing the distance matrices calculated for community composition with that of spatial distances associated with treatments within a site indicates the degree to which site‐level heterogeneity may contribute to community composition differences.

Finally, to compare plant community diversity within each restoration site for each seed mix treatment across time, we used non‐metric multidimensional scaling (NMDS) in three dimensions with a Bray–Curtis dissimilarity distance matrix derived from species community composition across each seed treatment and over time. We used NMDS, as it uses an ordination approach to summarize community composition across two‐axes, so that more similar communities cluster together (Ruiz‐Jaen & Aide, 2005). Temporal estimates included month and year of study incorporated into the ordination plots using the *envfit* function in the vegan package to tease apart the influence of seed mix treatment and time on plant communities' structure.

## 3 RESULTS

### 3.1 Seed viability

Six of the 14 species analyzed for seed viability had sufficient seed for a viability assessment. Variability in seed viability may impact how and whether individual species establish (presumed total number of individuals emerged per seed planted) within the first year following restoration (which may indicate hard seed or dormant seed). *Amorpha canescens* seed was 20% viable (16% germinable, 4% hard, and 0% dormant). Seed from *Anemone cylindrica* exhibited 82% viability with 75% germination, 0% hard seed, and 7% dormant. Seed from *Artemisia frigida* were 62% viable, with 25% germination, 0% hard, and 37% dormant. *Geum triflorum* seed had a total viability of 47% with 47% germination, and 0% hard, and 0% dormant. *Potentilla arguta* seed exhibited 88% viability, with 66% germination and 0% labeled as hard seed, 22% dormant seed. Finally, *Solidago rigida* seeds had a viability score of 54% with 44% of seed reaching germination, 10% labeled as hard seed, and 10% dormant seed.

### 3.2 Species community structure following restoration

**FIGURE 2:**
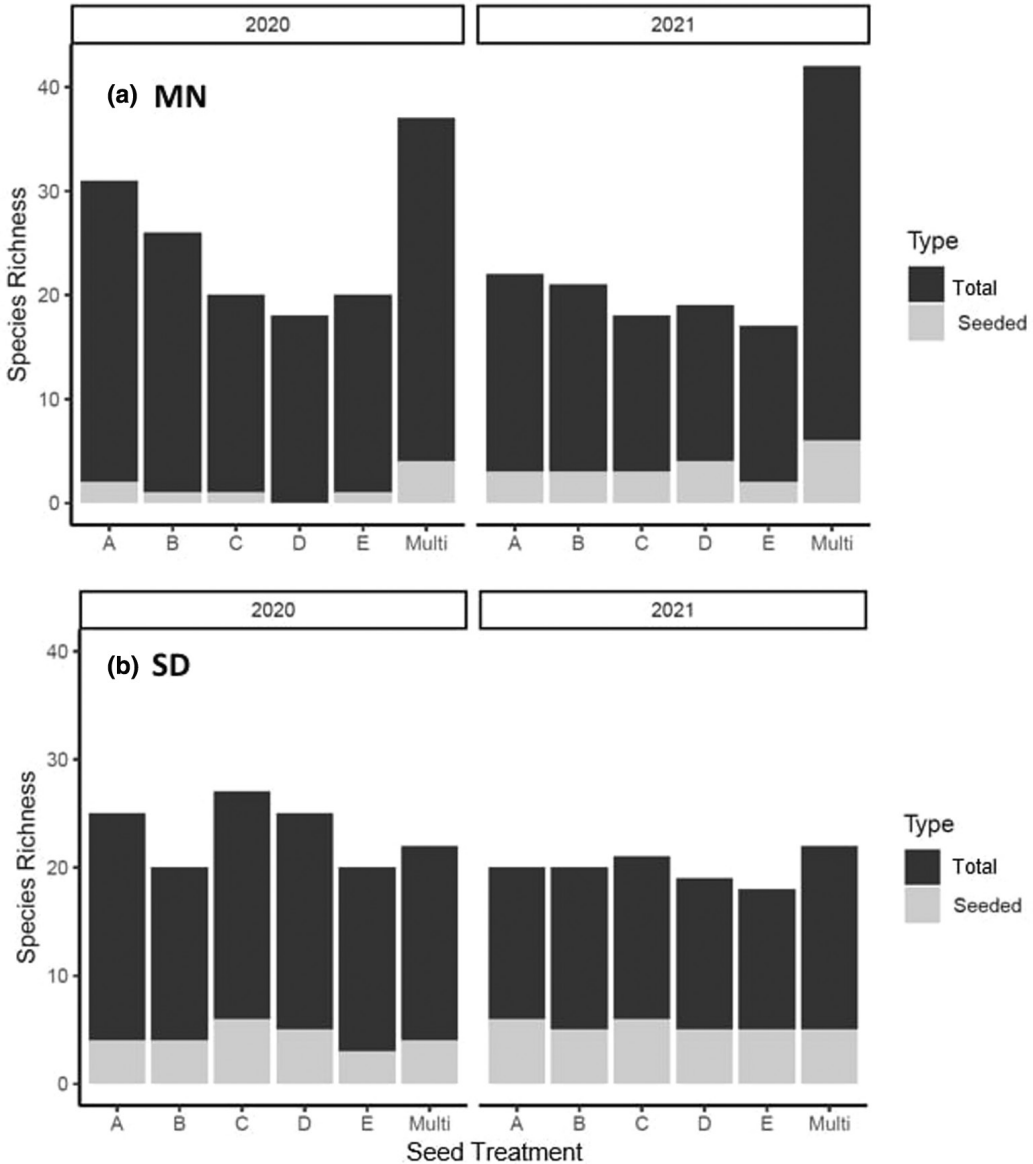
Comparison of seeded and total species richness within each seed treatment type across 2 years of data collection for MN experimental plots (a) and SD experimental plots (b). Total species richness was higher in MN than in SD, and the multiple source seed treatment had the greatest seeded species richness compared to single source seed treatments. Overall, seeded richness was greater within all SD plots compared to MN, but both sites had greater seeded species established within 2021 compared to 2020.

A mixture of seeded and non‐seeded species emerged at both the MN and SD restoration sites. At MN, seeded species emerged from all plots in the first growing season, except seed treatment “D”. Of seed mix treatment types, the multi‐source seed mix type “ABCDE” (“Multi”) had the greatest number of seeded species emerge, including *Echinacea angustifolia*, *H. maximilani*, and *Verbena hastata*. Across all seed treatments at the MN site, *H. maximilani* exhibited the greatest rate of emergence, followed by *Liatris punctata*. In the first year of observation, only four of the seeded species (*E. angustifolia*, *H. maximiliani*, *H. pauciflorus*, and *L. punctata*) established at MN and remained present in the second year of observation. In the second year, additional seeded species established at MN, including *A. canescens*, *A. cylindrica*, *A. frigida*, *Drymocallis arguta*, *G. triflorum*, and *S. rigida*. The total number of seeded species that were established was greater in the second year. Across seed mix treatments, the multi‐source treatment continued to exhibit the greatest seeded species richness (Figure 2a). Overall, following the second year, *A. cylindrica*, *H. maximiliani*, and *S. rigida* exhibited most individuals emerging across multiple seed treatments.

At the SD site, seeded species emerged from all plots in the first growing season. The multi‐source seed mix “ABCDE” and the single‐source seed treatment “C” had the greatest number of seeded species emerge overall in the first year. This included the emergence of *H. maximiliani* and *S. rigida*, observed across all seed mix treatments, followed by *Ratibida columnifera* and *Dalea purpurea*. In total, only six unique seeded species established at the SD site in the first year (*Bouteloua curtipendula*, *B. gracilis*, *D. purpurea*, *H. maximiliani*, *R. columnifera*, and *S. rigida*). In the second year, all six species were again observed plus *A. cylindrica*, *E. angustifolia*, *H. pauciflorus*, *L. punctata*, and *D. arguta*. Overall, in the second year, *B. curtipendula*, *H. maximiliani*, *R. columnifera*, and *S. rigida* exhibited the most individuals emerging across multiple seed treatments.

Throughout the study non‐seeded species largely dominated at both restoration sites across seed mix treatments (Figure 2). At the MN site, the most common species observed within our experimental restoration plots were *Ambrosia psilostachya*, *Melilotus* sp., *Panicum capillare*, *Poa pratensis*, *Oxalis stricta*, *Trifolium repens*. At the SD site the most common species within our experimental restoration plots were *A. absinthium*, *Bromus inermis*, and *P. pratensis*.

**FIGURE 3:**
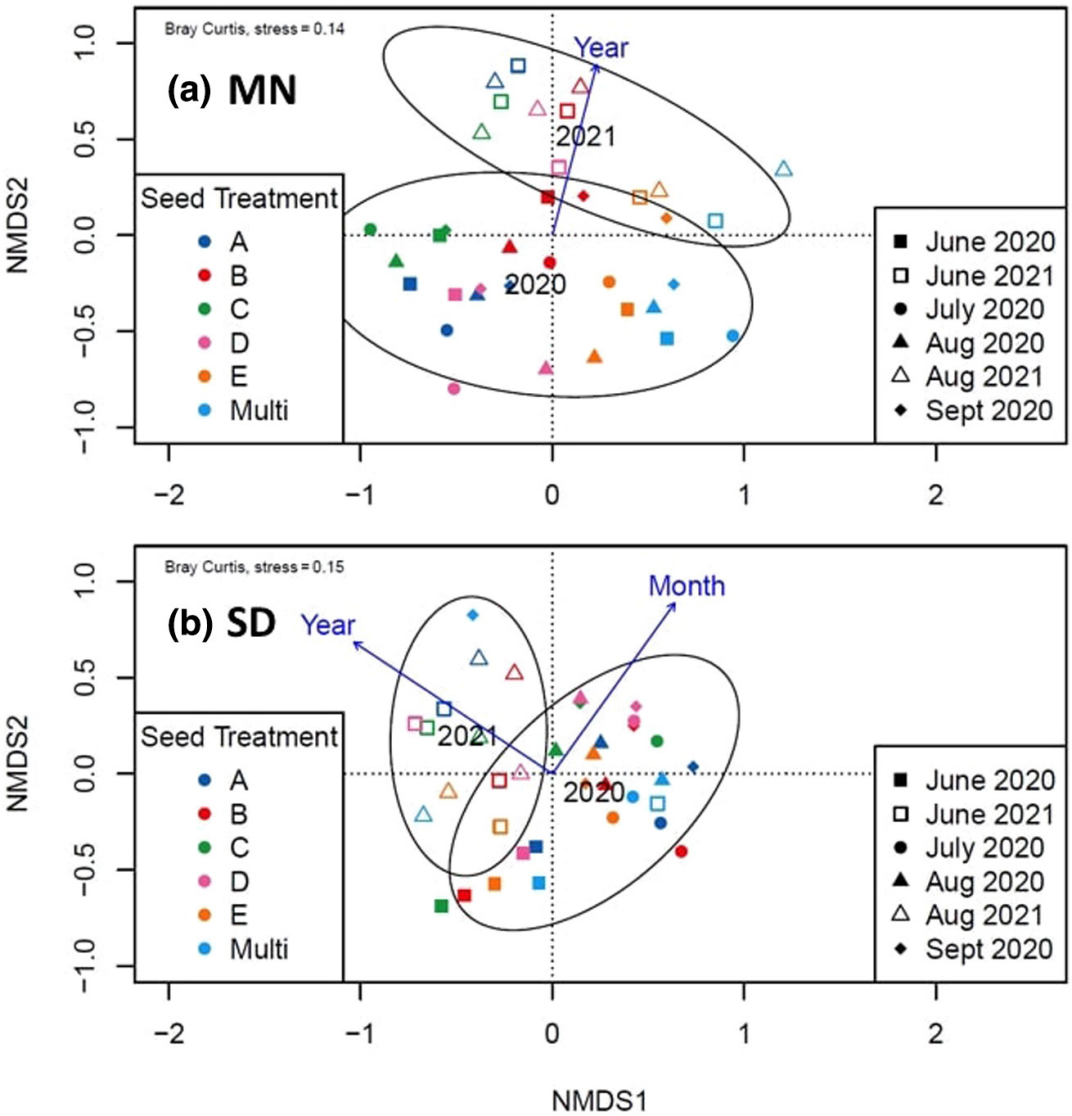
Nonmetric Multidimensional Scaling with Bray – Curtis dissimilarity graphs of the community establishment similarities within seed treatment and across sampling years. Both (a) MN seed treatment plots and (b) SD seed treatment plots are grouped by year. Colors indicate seed treatments and shapes indicate month of data collection. Ellipses are 95% confidence intervals. Year and month were evaluated as vectors influencing community similarity. Arrows indicate direction and length shows the degree of separation. Only those vectors that significantly influenced community composition are shown in the ordination space.

To test the effect of seed mix treatment on community composition at the MN site, we first evaluated whether uneven sample size across replicate seed mix treatments influenced dispersion of values related to community composition. Beta‐diversity did not significantly differ between seed treatments (*F* = 0.63, *p* = .671) this suggests that unequal seed treatment size did not influence potential differences across seed treatments. Next, we used a PERMANOVA to evaluate plant community differences between seed mix treatments across the 2 years (Oksanen et al., 2022). We observed significant community‐level differences across seed mix treatments in 2020 (pseudo‐*F* = 2.91, *p* = .002) and 2021 (pseudo‐*F* = 5.88, *p* = .001; Table 1). Although seed mix treatment did influence community composition at the MN site, very few seeded species established across seed mix treatments, suggesting that the differences observed were likely driven by site‐level differences associated with spatial heterogeneity in the presence of non‐seeded species (Figure 3a). These results point toward the importance of spatial heterogeneity within a restoration site and its influence on early post‐restoration emergence patterns.

**TABLE 1:** PERMANOVA results to evaluate the role of seed mix treatment to species composition at the Northwestern Minnesota (MN) restoration site in the Agassiz Beach Ridges in 2020 (a) and 2021 (b).

	Df	SS	MS	Pseudo *F*	*R* ^2^	*p*
(a) MN community composition subset for 2020						
Seed mix treatment	5	1.576	0.315	2.905	.708	.002***
Residuals	6	0.651	0.109	0.292		
Total	11	2.227	1			
(b) MN community composition subset for 2021						
Seed mix treatment	5	1.379	0.276	5.875	.830	.001***
Residuals	6	0.282	0.047	0.170		
Total	11	1.661	1			

*Note*: Significant *p*‐values *p* < .05 denoted by *** indicates a significant effect of the variable or interaction on species composition.

Mantel tests evaluated if there was a correlation between our species community composition as a distance matrix compared to the spatial distance matrix across seed treatments within the restoration site. This test evaluated the influence that spatial heterogeneity in restoration site across seed mix plots may have on community composition over time. Within the MN site, only August of 2020 had significant results (*r* = .569, *p* = .05), indicating a potentially positive effect of seed treatment location within the site on community composition, but only during that late season period. To further visualize differences in plant communities we used an NMDS with Bray–Curtis dissimilarity matrix with month and year of data collection fit to the ordination plot (Figure 3a). This ordination approach indicated plant communities clustered according to seed mix treatment with little overlap in community composition across the 2 years. Consistent with our previous experiences, seed mix treatment (or site location) influences community composition, and these differences persisted across both years at MN.

As above, we first tested for dispersion in our dataset to determine whether uneven sample size contributed to community composition differences at SD. No significant influence of dispersion was observed within the SD site (*F* = 2.42, *p* = .087), indicating beta diversity within seed mixes was not significantly different. This suggests that differences observed using PERMANOVA are likely not a result of unequal sampling between single and five source seed mix plots. However, PERMANOVA results indicated there were significant community‐level differences based on seed mix treatments in 2021 (pseudo‐*F* = 2.56, *p* = .001; Table 2). Like the MN site, seeded species establishment at the SD site was low; therefore, we cannot rule out the possible effect of seed mix treatment locations across the site. However, Mantel tests to compare spatial distance across treatment locations with species composition differences were not significant across any period. This indicates that spatial heterogeneity across the SD site likely had minimal effect on community composition for seed mix treatments. Rather, the limited differences observed in community diversity are likely due to seed mix treatments. NMDS indicated overlap in community composition across years of reflected clusters associated with months of observation (phenology), with limited clustering associated with seed treatment (Figure 3b). This suggests that community composition changes over time at the SD site were most strongly influenced by the growing season (months and years), as opposed to seed mix treatment. The impact of year suggests that temporal compositional change in diversity may play an important role early in the development of plant communities post‐restoration.

**TABLE 2:** PERMANOVA results to evaluate the role of seed mix treatment to species composition at the South Dakota (SD) restoration site in the Missouri Coteau in 2020 (a) and 2021 (b).

	Df	SS	MS	Pseudo *F*	*R* ^2^	*p*
(a) SD community composition subset for 2020						
Seed treatment	5	0.593	0.119	1.287	.518	.237
Residuals	6	0.553	0.092	0.482		
Total	11	1.146	1			
(b) SD community composition subset for 2021						
Seed treatment	5	0.600	0.120	2.562	.681	.001***
Residuals	6	0.281	0.047	0.319		
Total	11	0.881	1			

*Note*: Significant *p*‐values *p* < .05 denoted by *** indicates a significant effect of the variable or interaction on species composition.

## 4 DISCUSSION

Establishing seed mixes that consider genetic diversity is critical to ensuring communities are resilient to change. Using the number of seed sources as a proxy for genetic variation, we compared single and multi‐source seed mix treatments to plant community composition across two sites. Restoration sites were largely dominated by non‐seeded species, with limited emergence of seeded species the first 2‐years post‐restoration. These observations are consistent with previous restoration studies, which indicate that non‐seeded species may dominate restored environments during the first several years following restoration (Kaul & Wilsey, 2021; McLachlan & Knispel, 2005). Interestingly, on average, the multi‐source seed treatments exhibited greater diversity of seeded species across both sites over time, suggesting that multiple sources can be important to early post‐restoration diversity. Despite this, site history likely had a greater impact on early plant establishment and community composition relative to seed mix treatment across both sites. While early observations suggest that there may be limited influence of seed mix treatment on community diversity, longer term observations are needed to monitor plant establishment over time.

### 4.1 Seed viability and dormancy may impact early emergence

Although non‐seeded species were expected in the first year, variation in seed viability within our seed mixes (ranging from 20% to 88% at MN) may have impacted first year emergence. Between 7% and 37% of seeds evaluated were hard and dormant, which may indicate individuals will germinate in subsequent years provided environmental conditions are favorable (Jensen, 2004). This aligns with the greater number of seeded species observed in the second year of observation. In addition, seed predation and seedling herbivory may have reduced establishment success during the first year. Herbivore disturbance can influence the dispersal, establishment, and dominance of non‐seeded species through selective herbivory on native plant species (Howe & Brown, 2000). At the MN site, observations of the 13‐lined ground squirrel (*Ictidomys tridecemlineatus*) and evidence of gophers (*Thomomys* sp.) suggest that herbivory may have impacted the seeded plant community. There were no squirrels or gophers observed in SD; however, other parts of the property have populations of Richardson's ground squirrels (*Urocitellus richardsonii*) suggesting that they could have been present at SD. Our study design was aimed to mimic natural restoration practice and did not take measures to actively exclude mammals from the restoration sites. Instead, we used approximately five times the standard seeding rate for each seed mix. Higher seeding rates are often used to mitigate potential effects of seed viability and herbivory on seedling establishment to increase restored plant densities (Applestein et al., 2018).

### 4.2 Seeded and non‐seeded species richness across restoration sites

We compared species richness of seed mix treatments across the two restoration sites. On average, multi‐source seed mixes were associated with greater seeded species richness across sites over time (Figure 2). At the MN site, multi‐source seed mixes exhibited greatest species richness across both years. However, species richness was only greatest in the second year for the SD site. In MN, in the first year of observation, non‐seeded species established at seven times the number of seeded species. Additional seeded species established in the second year, suggesting that variability in viability and dormancy of seeds may have influenced seeded species presence early post‐restoration (Figure 2). The SD restoration site exhibited approximately four times the number of non‐seeded species relative to seeded species across both years. However, community composition across the different seed mix treatment types was more uniform relative to differences observed at the MN site (Figure 2). In general, these rates are consistent with previously observed rates of seeded versus non‐seeded plants establishing early in grassland restoration experiments (Martin et al., 2005). This is despite the fact that preferred seedbed preparations, such as clean‐till, were not used to limit non‐seeded species colonization in early years post‐restoration (Dixon et al., 2017). However, they may also reflect the role of site history and heterogeneity in the restoration environment for community establishment.

Plant community composition may be influenced by several factors, including the biotic and abiotic factors associated with a site, species dispersal ability, local species competition and more (reviewed in Zobel, 2016). Given this, we expected site history could influence community composition across restoration sites and seed mix treatments. For MN, the multi‐source seed mix treatment (ABCDE) exhibited the greatest total diversity throughout the observational period. However, the multi‐source seed treatments were planted on the portion of the restored site that was once a golf course and near a remnant mesic area with surrounding woody vegetation. This may have contributed to microsite differences relative to other treatment sites. Several species, including *Achillea millefolium*, *Plantago major*, *P. annua*, and *Salix interior* established solely within this treatment and were persistent within nearby woody vegetation. Given these observations, richness observed in the multi‐source seed treatment may reflect diversity within the local seed bank over that of seed mix treatment. Indeed, this treatment was different from all other seed mix treatments, except seed mix “E,” which was compositionally more similar during later seasonal months. Given the spatial proximity of the “ABCDE” and “E” treatments (Figure 1), similar communities likely arose due to local site conditions, including belowground nutrient resources and seed banks. To account for geographic proximity, we used a Mantel's test to evaluate the effect pairwise spatial differences across treatments may have on community establishment. The significant location effect observed during the later season (August) suggests that, although seed mix treatments appear different, microsite differences may be confounding differences observed across the restored site (Figure 3a).

Overall, total sown species richness was greater at the SD site. Despite this, there were no differences in plant community composition associated with seed mix treatment (Figure 2). The total increase in seeded species richness could indicate less competition from non‐seeded species or that seeded species already existed within the soil seed banks. The Mantel's test suggests that there is no relationship between spatial distance between seed mix treatments and species' composition, indicating the restoration site is largely homogeneous. In fact, growing season appeared to have the strongest influence on plant community structure (Figure 3b). Community diversity followed a similar trajectory across all seed mix treatments, suggesting that temporal effects associated with growing season were important irrespective of seed mix treatment. Seasonal variation may drive community composition through niche differentiation, which may allow for a higher diversity of plants to coexist in the same space at different time points (Dolezal et al., 2019; Schofield et al., 2018).

Across sites, early emergence patterns and plant community composition largely reflected land‐use histories despite seed mix treatments. The SD site was an old agricultural field with ongoing active management used for hay production. Similar land‐use history across the site likely homogenized the above‐ and below‐ground plant community, which was dominated by smooth brome (*B. inermis*) and alfalfa (*Medicago sativa*). These dominant species likely influenced the seed bank, further reducing richness and diversity within the non‐seeded species community (Bekker et al., 1997). In contrast to the homogeneity observed at SD, the land‐use history at MN was more heterogeneous. This may have contributed to significant differences associated with seed mix treatment. Single‐source seed treatments A, B, and C were established on a portion of the site that was once planted with brome and alfalfa for haying. However, seed mix treatments D, E, and the multiple‐source mix ABCDE were established on a portion of the site that was a golf course until 2015 (Figure 1). Combined, variation in land use history, variation in soil nutrient profile, and associated impacts to the seed bank point toward substantial heterogeneity across the restoration site that may have influenced early emergence patterns following application of seed mix treatments (Williams & Houseman, 2014).

### 4.3 Species life history influences emergence following restoration

Seeded species that emerged in our experiment were those that have evolved traits that provide competitive advantages in grassland ecosystems, such as rhizomatous root systems (Dickson & Busby, 2009; Mangan et al., 2011) or mutualistic relationships that promote establishment (Busby et al., 2011). For example, *H. maximiliani* is a widespread perennial forb native to prairies in the United States and Canada. *H. maximiliani* readily established at both sites across seed treatments and is often found in remnant and restored prairies as a sub‐dominant or dominant species (Braasch et al., 2021; Dickson & Busby, 2009). Previous studies have found that *H. maximiliani* is often one of the most productive forb species within plant communities due to its rhizomatous root system that creates a spreading pattern for nutrient uptake and a thick sprouting stem that leads to increased biomass production and vegetative cover (Dickson & Busby, 2009; Mangan et al., 2011; McKenna et al., 2019). *Ratibida columnifera* was another common perennial species to establish at SD and across seed treatments. In previous experiments, *R. columnifera* has been observed to have high first‐year survival and a lifespan of around 3 years, and it may negatively impact the abundance of other forbs (Dickson & Busby, 2009; Lauenroth & Adler, 2008). The competitive advantage of *R. columnifera* may be attributed to a prominent taproot and a strong positive relationship with arbuscular mycorrhizal fungi that aids nutrient uptake and growth (Busby et al., 2011). Both species are native to our study region and likely existed within the seed bank at both sites. However, *H. maximiliani* was not observed at either site outside of the plots. *R. columnifera* was present at the MN site, but was not included in the experimental seed mixes. Identifying those species that establish early during restoration may help inform design of future seed mixes designed intended to limit non‐native species establishment, ensuring long‐term restoration success.

Although a primary goal of this study was to quantify diversity for seeded species established across seed mix treatments, presence of non‐seeded species is also important to consider. In a previous study, Kaul and Wilsey (2021) noted that abundance of non‐seeded weedy species was the strongest predictor of species richness and diversity in grassland restorations, regardless of restoration age. The most common non‐seeded species to establish within our communities were introduced species, including cool‐season grasses *B. inermis* and *Poa pratensis*. These species typically outcompete natives for resources, including both nutrient and light availability (reviewed in D'Antonio & Meyerson, 2002). *P. pratensis* establishes early in the spring before many native forbs, which may provide a competitive advantage over native species (DeKeyser et al., 2015). *B. inermis*, a commonly planted pasture grass, also establishes readily in the spring and forms a monoculture through its rhizomatous root system (Stotz et al., 2019). *B. inermis* often outcompetes and displaces native species, leading to plant community homogenization (DiAllesandro et al., 2013; Stotz et al., 2019). The prevalence of these well‐known invasive species within our treatments despite our pre‐seeding site preparation indicates our effort had limited impact. Considering how easily these species establish suggests that more effort may be needed to shift these communities back to native species (Martin & Wilsey, 2014). Genetic variation may increase the diversity of genotypes that establish following restoration, increasing the probability of producing a self‐sustaining, persistent population that can evolve over generations (Hofner et al., 2022). In this way, genetic variation within seeded species may mitigate some of the negative impacts of invasives.

### 4.4 Early emergence exhibits limited sensitivity to seed treatments post‐restoration

Single versus multi‐source seed mixes appeared to have limited impact on community composition diversity in the first years following restoration. Our results suggest that early emergence and diversity is largely influenced by land‐use history. Early emergence can be largely insensitive to seed mix treatment if non‐seeded species in the seedbank outcompete seeded species during establishment. Previous studies have shown that first year emergence positively influences seeded species abundance and richness several years post‐restoration (Applestein et al., 2018; Geaumont et al., 2019). Thus, while there is some evidence to suggest that seed source diversity may enhance richness and diversity of established species, long‐term assessments over multiple years will be necessary to quantify the full impact of seed mix type to community diversity and restoration success over time. In addition, future work should include a genetic analysis of populations in single and multi‐source seed mixtures to quantify genetic variation applied within seed mix treatments. Combined, genetic analysis and longer term monitoring of seed mix treatments will provide information needed for land managers to establish seed sourcing guidelines critical to restoration in a changing environment.

## AUTHOR CONTRIBUTIONS

**Jessica Lindstrom:** Conceptualization (equal); data curation (lead); formal analysis (lead); investigation (lead); methodology (lead); writing – original draft (lead); writing – review and editing (equal). **Marissa Ahlering:** Conceptualization (supporting); formal analysis (supporting); funding acquisition (lead); investigation (lead); project administration (lead); resources (supporting); writing – original draft (supporting); writing – review and editing (supporting). **Jill Hamilton:** Conceptualization (lead); formal analysis (supporting); funding acquisition (lead); methodology (supporting); project administration (lead); resources (supporting); writing – original draft (supporting); writing – review and editing (supporting).

## CONFLICT OF INTEREST STATEMENT

The authors declare no conflict of interest.

## Supporting information


Data S1:

## Data Availability

All data are provided openly available as supplemental tables associated with this manuscript.
