# Supplementary material for: Seed sourcing for climate‐resilient grasslands: The role of seed source diversity during early restoration establishment
Source: Ecol Evol. 2023 Nov 21;13(11):e10756. doi: 10.1002/ece3.10756 (PMC10663101; doi:10.1002/ece3.10756)
Supplement: Supplementary file 1 — Data S1: [file ECE3-13-e10756-s001.docx]

Supplemental Data Tables
